# Statistical methods to model and evaluate physical activity programs, using step counts: A systematic review

**DOI:** 10.1371/journal.pone.0206763

**Published:** 2018-11-02

**Authors:** S. S. M. Silva, Madawa W. Jayawardana, Denny Meyer

**Affiliations:** Department of Statistics, Data Science and Epidemiology, Swinburne University of Technology, Hawthorn, Victoria, Australia; Universidad de Tarapaca, CHILE

## Abstract

**Background:**

Physical activity reduces the risk of noncommunicable diseases and is therefore an essential component of a healthy lifestyle. Regular engagement in physical activity can produce immediate and long term health benefits. However, physical activity levels are not as high as might be expected. For example, according to the global World Health Organization (WHO) 2017 statistics, more than 80% of the world’s adolescents are insufficiently physically active. In response to this problem, physical activity programs have become popular, with step counts commonly used to measure program performance. Analysing step count data and the statistical modeling of this data is therefore important for evaluating individual and program performance. This study reviews the statistical methods that are used to model and evaluate physical activity programs, using step counts.

**Methods:**

Adhering to PRISMA guidelines, this review systematically searched for relevant journal articles which were published between January 2000 and August 2017 in any of three databases (PubMed, PsycINFO and Web of Science). Only the journal articles which used a statistical model in analysing step counts for a healthy sample of participants, enrolled in an intervention involving physical exercise or a physical activity program, were included in this study. In these programs the activities considered were natural elements of everyday life rather than special activity interventions.

**Results:**

This systematic review was able to identify 78 unique articles describing statistical models for analysing step counts obtained through physical activity programs. General linear models and generalized linear models were the most popular methods used followed by multilevel models, while structural equation modeling was only used for measuring the personal and psychological factors related to step counts. Surprisingly no use was made of time series analysis for analysing step count data. The review also suggested several strategies for the personalisation of physical activity programs.

**Conclusions:**

Overall, it appears that the physical activity levels of people involved in such programs vary across individuals depending on psychosocial, demographic, weather and climatic factors. Statistical models can provide a better understanding of the impact of these factors, allowing for the provision of more personalised physical activity programs, which are expected to produce better immediate and long-term outcomes for participants. It is hoped that this review will identify the statistical methods which are most suitable for this purpose.

## Introduction

According to the World Health Organization (WHO), world-wide obesity has doubled since 1980 [1]. In most countries in the world being obese or overweight is more likely to contribute to mortality than being underweight. In 2016, more than 1.9 billion adults 18 years or over (i.e. 39% of the world’s adults) were overweight and 600 million (13%) were obese [1]. When considering child populations under the age of 5 years, 41 million children were overweight or obese in 2016. However, obesity is preventable through various measures [1–3]. The main reason for being overweight or obese is an energy imbalance between consumed calories and expended calories. This may be due to greater intake of energy-dense foods which are high in fat, or, to physical inactivity. A common consequence of being overweight or obese is a higher risk of noncommunicable diseases such as cardiovascular diseases, diabetes, musculoskeletal disorders and some cancers (including breast, liver and ovarian cancers) [1].

Through its relationship with being overweight/obese, inactivity is bad for health, whereas physical activity has significant health benefits. Physical activity has been defined as “*any bodily movement produced by skeletal muscles that requires energy expenditure including activities undertaken while working, playing, carrying out household chores, traveling, and engaging in recreational pursuits*” [3] and even moderate intensity physical activity has significant health benefits as long as it is regular [1–3].

The WHO recommends different levels of physical activity for different age groups [3]. For cardiorespiratory health benefits, all activities need to occur in bouts with a duration of at least ten minutes [3]. Global statistics show that 23% of adults aged 18 and over are not sufficiently physically active [3] and high income countries have higher physical inactivity ratios than lower income countries. As a result of these statistics, physical activity programs have been introduced in many countries with the goal of improving the physical activity of participants. These programs should also help the participants to improve their quality of life and reduce the risk of noncommunicable diseases.

Different types of activity program are used to target populations with differing characteristics. Several systematic reviews discuss the effectiveness of school-based physical activity programs [4–7]. For example, the systematic review in [8] suggests that there is a positive effect on behavior and physical status resulting from ongoing school-based physical activity interventions. The next class of physical activity programs are work-based programs because people spend so much time at work [9–14]. Lastly, community-based physical activity programs usually focus on a defined group of participants (e.g. retired senior people), in order to promote the importance of physical activity for improving quality of life [15–21]. Assessing the effectiveness of these programs has been a popular undertaking among researchers. However, there has been limited research conducted on identifying the important factors for the personalisation of these programs. In order to deliver a more personalised program, there should be an acceptable way of grouping participants and a systematic approach for adjusting the program format appropriately for individual groups. This personalisation is expected to improve the program effectiveness for everyone.

In this systematic review the statistical modeling techniques used to identify suitable factors for program personalisation will be considered. Walking and running are the most popular physical activities that are included in almost every physical activity program. For this reason, we decided to use step counts, tracked through a device such as a pedometer or accelerometer, for assessing the physical activity levels of participants. The majority of the studies using accelerometers or pedometers collected raw activity counts data, have focused on a specified scope of kinematics, predicting abnormal human behavior and disorders by utilizing various kinds of supervised and unsupervised data mining methods. However, in this systematic review we focus only on the journal articles that consider step counts as a specific study variable, which is collected through a tracking device and used in some form of statistical modeling to assess the program outcomes. It is expected that these articles will help the researchers to identify appropriate statistical models for evaluating and personalising physical activity programs utilizing step count data. Further we hope that the findings of this systematic review will help to create more effective physical activity programs for promoting healthier lifestyles in the future.

## Methods

### Registration and protocol

This systematic review was reported in accordance with the preferred reporting items for systematic review and meta-analysis protocols (PRISMA-P) 2015 statement [22]. This review has been registered with PROSPERO [23] under the record number CRD42017076786 on 31^st^ of October 2017. The protocol for this review has been published in protocols.io in order to enhance the reproducibility of the results. The protocol can be accessed from: http://dx.doi.org/10.17504/protocols.io.tsvene6.

### Literature search strategy and assessment

The study considered any intervention which relates to physical activity and step counts. To find the relevant research articles, an individualized search strategy was built for the PubMed, Web of Science and PsychINFO databases. This literature search was limited to the journal articles which were published from January 2000 to August 2017. The above databases all show an increasing trend in physical activity research from 2000 onwards, making research prior to this date of less relevance. Moreover, the usage of pedometers or accelerometers in physical activity research has also particularly increased since 2000 due to technological advancements. Because of time constraints it was decided to include only articles published in English. It was decided to use individualized key words in the search strategy for the above mentioned databases, in order to extract more relevant articles. It should be noted that for Pubmed and PsycINFO, some of the key words were MeSH terms and this helped to ensure more relevant articles. However, for the Web of Science database, it was decided to use defined key words, since this database does not have a library indexed language. The following list denotes the series of key words used for each database.

### PubMed

accelerometer OR pedometer OR step count* OR “Fitness Trackers”[Mesh] AND statistic* OR statistical model* OR quantitative OR “Models, Statistical”[Mesh] OR “Data Mining”[Mesh] OR “Data Interpretation, Statistical”[Mesh] AND “Exercise”[Mesh] OR “physical activity” OR fitness OR Program* OR intervention.

### PsycINFO

accelerometer OR pedometer OR step count* OR “Fitness Trackers” AND statistic* OR statistical model* OR quantitative OR “Statistical Analysis”[Index Term] OR “Data Mining”[Index Term] OR “Data Interpretation” AND “Exercise”[Index Term] OR “physical activity” OR fitness OR Program* OR intervention.

### Web of Science

accelerometer OR pedometer OR step count* OR “Fitness Trackers” AND statistic* OR statistical model* OR quantitative OR “Data Mining” OR “Data Interpretation” AND “Exercise” OR “physical activity” OR fitness OR Program* OR intervention.

### Inclusion and exclusion criteria

To select articles better matched to the objective of this systematic review, five main inclusion criteria were developed. Articles were selected if (i) the study included data taken from a physical activity program or an intervention; (ii) the data collection did not impact the usual natural lifestyle of the participants (e.g. studies where data was collected through walking on a treadmill or from a walking test were excluded); (iii) the study needed to be carried out with at least one group of healthy participants; (iv) the study needed to include a study variable related to “step counts” and this data needed to be collected using a device such as a pedometer or accelerometer; (v) the study needed to carry out a quantitative analysis using “statistical modeling”.

### Selection process and data extraction

**Fig 1:**
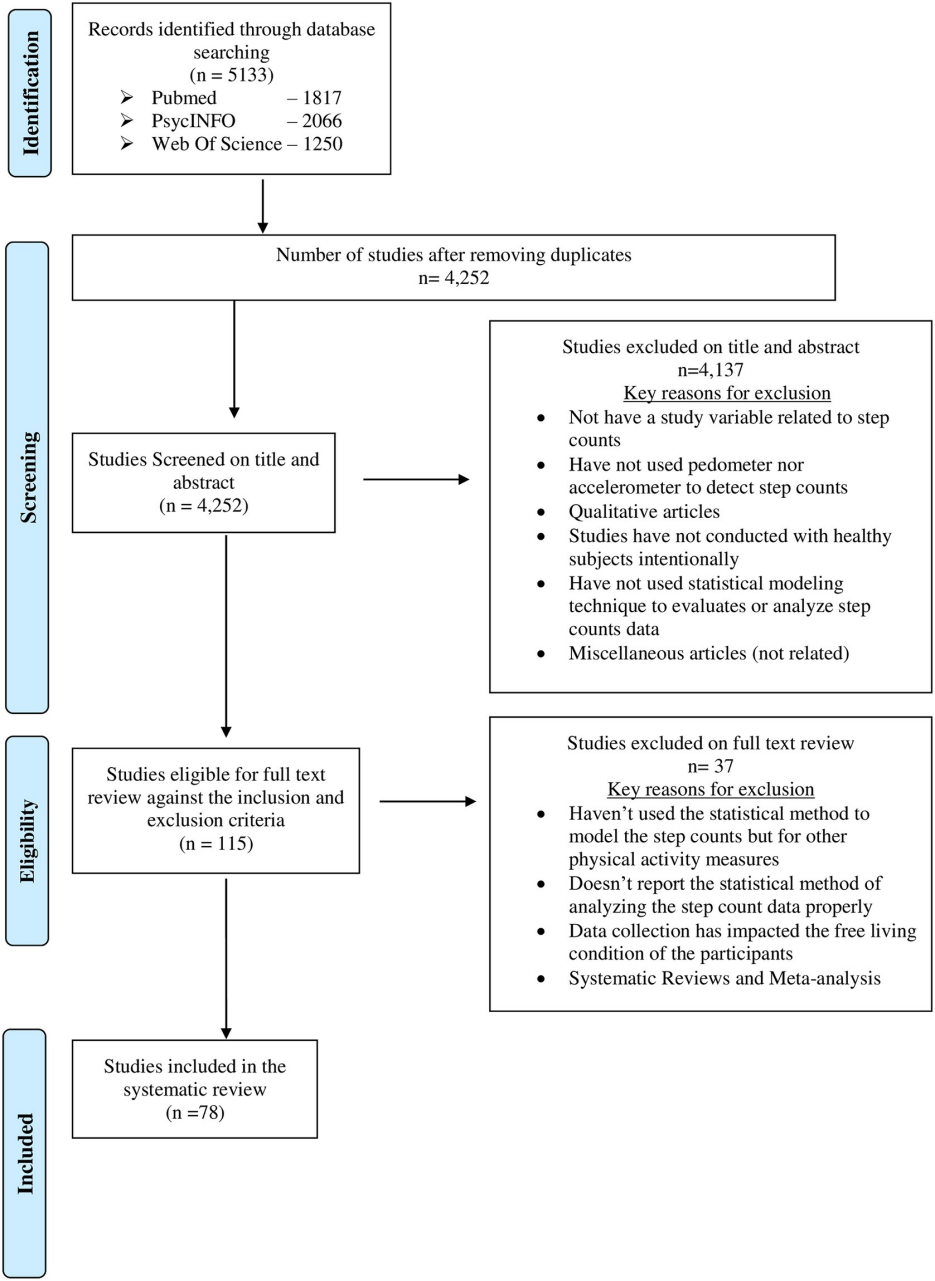
PRISMA flow diagram.

The initial database search was able to identify a total of 5133 articles which included 1817 articles from Pubmed/Medline, 2066 articles from PsycINFO and 1250 articles from Web of Science. These articles were uploaded to EndNote software and 881 duplicates were removed by the first author. The other two authors were then given online access to view the results. The first two authors then independently screened the titles and abstracts against the inclusion criteria and shortlisted the most relevant articles. The articles which had been accepted by both reviewers were shortlisted and the articles which had questionable eligibility were directed to the third reviewer for resolution. This process shortlisted 157 articles for a full text review, out of which the team agreed on 78 unique articles for inclusion in this review. The following PRISMA flow chart (Fig 1) outlines the above search and review process.

### Quality assessment

The Downs and Black assessment tool [24] for health care interventions was used to measure the study quality of the included articles. This tool is appropriate for assessing both randomized and non-randomized studies of health care interventions. This checklist consists of 27 items distributed among five sub scales, namely; reporting (10 items), external validity (3 items:), bias (7 items:), confounding (6 items:) and power (1 item:). According to the tool, the maximum score that a study can obtain is 32, with higher scores indicating better quality. In this review the Downs and Black scores have been categorized as follows; high quality (22-25), moderate quality (18-21) and low quality (14-17).

### Taxonomy for statistical techniques

There are many different statistical techniques that can be used to address the aims and objectives of a study with the available data. Therefore, the “guide for choosing among statistical techniques” published in [25] has been used to classify the various techniques found in the shortlisted articles.

## Results

A total of 78 articles were included in this systematic review, representing more than 20 different countries, with more than one country providing the data for some of the articles [26]. Most of the articles were from the United States of America (27), followed by the United Kingdom (8), Canada (7) and Australia (6) and the sample sizes for these studies ranged from 10 to 11,658. There were observational study designs (71%) as well as experimental study designs (29%), comprising school, work and community based physical activity interventions. Due to the variability in the included studies in terms of study design, sample size, intervention, objectives and statistical methods for analysing the data, this systematic review will provide a qualitative review (rather than a meta-analysis) on the reported statistical methods used.

Thirteen articles included in the review used more than one statistical modeling technique in analysing the step counts [4, 13, 27–37]. It was noted that 60% of the articles used general linear models, while 24% of the articles used generalized linear models, 10% of the articles used multilevel modeling, 4% used structural equation modeling and 2% of the articles used generalized estimating equations. Among the articles which used general linear models, analysis of variance (ANOVA) and other related techniques were most preferred (60%), followed by simple linear regression techniques (40%). Under generalized linear models, logistic regression (including ordinal logistic regression and nominal logistic regression) was the most preferred (91%) method, followed by the exponential regression and log-binomial regression methods. Contrary to expectation no studies with time series models for step counts were found.

The included studies can mostly be divided into two categories depending on the study objective and analysis, studies that analysed the “effect *ON* step count” and studies that analysed the “effect *OF* step counts”. The majority of the studies (76%) were categorized as “effect *ON* step counts” whereas 24% of the studies were categorized as “effect *OF* step counts”. These two main categories were further sub-divided, depending on the main factors and the scope of the study considered within the analysis. Tables 1 and 2 show a summary of the statistical methods grouped under these categories.

**Table 1:** Statistical models used in analysing step count data in relation to the scope of the article: Effect *ON* step counts.

Statistical Modeling Procedure	Family, Community and Sociodemographic	Health Related	Intervention	Personal and psychological behavior	Weather and demographic	Other
General Linear Models	12	0	18	3	2	7
Generalized Liner Models	9	0	1	2	0	0
Multilevel Models including Generalized Estimating Equations	1	1	6	1	2	0
Structural Equation Modeling	0	0	0	4	0	0
TOTAL (69)	22	1	25	10	4	7

**Table 2:** Statistical models used in analysing step count data in relation to the scope of the article: Effect *OF* step counts.

Statistical Modeling Procedure	Health Related	Other
General Linear Models	11	2
Generalized Liner Models	10	0
TOTAL (23)	21	2

Clearly, general linear models are popular among the authors in almost every category except for “personal and psychological behavior”. Structural equation modeling is the most preferred method of analysis under this category, appearing nowhere else. Multilevel modeling is only used to analyse the “effect *ON* step counts”. Interestingly health related articles are the most likely to consider the effect *OF* step counts whereas articles considering the effect *ON* step counts consider a much broader range of topics. Table in S1 Table summarized the data extracted from the shortlisted articles.

### Risk of bias assessment

A risk of bias assessment was carried out for all the 78 studies using the Downs and Black study quality assessment tool. The quality scores ranged between 14 and 25 with a mean score of 19.7. Four studies were categorized as high quality (22-25), 63 studies as moderate quality (18-21) and 11 studies as low quality (14-17).

### Family, community and sociodemographic scope

It was noted that 31% of the family, community and sociodemographic studies were classified as “effect *ON* step counts”. Out of these studies 55% used general linear models while 41% used generalized linear models, with one using multilevel modeling. The majority of the general linear model and generalized linear model studies had a cross sectional design (75% and 89% respectively), while the study which used multilevel modeling had a cluster randomized design.

The majority (58%) of the general linear model analyses used ANOVA or linear regression. Several socio demographic studies examined the significance of predictor variables such as age, sex, marital status, employment status and race, as well as the interactions of these variables, in terms of physical activity [9, 33, 38–42]. These studies considered participant samples ranging from 8 years to 83 years. It was found that for several studies age and gender were significant predictors of physical activity [33, 38, 40]. Moreover it was also found that the use of new technological devices such as physical activity watches, was more likely to increase peoples’ physical activity [42]. Ordinary least squares regression models were also used to examine the association between income and physical activity [43], and multivariate linear regression models were used in order to test the relationship between parental beliefs and support for the physical activity levels of children, measured using pedometers [34, 44]. However, the effect of climate and weather and neighbourhood environment are also important determinants of physical activity levels. The influence of neighborhood walkability has been tested by estimating the mean differences in steps per day across neighborhoods with different walkability scores, finding no significant relationship [45]. A similar type of analysis has been performed using gender stratified multilevel modeling to analyse the association between neighborhood safety and pedometer measured physical activity, allowing for within neighbourhood clustering [46]. This study used a randomized cluster design with 12 urban public housing communities as the primary sampling units and individuals within these communities as secondary sampling units. The authors have considered day-time safety and night-time safety separately.

All the studies which used generalized linear regression methods have used logistic regression models to investigate significant sociodemographic effects for the achievement of various levels of physical activity [33, 47–51]. For these analyses variables such as city of residence, dog ownership, motor vehicle ownership, education level, occupation type, self rated income level, usual daily occupational or domestic activity level and transport mode to work have been considered as predictors. It was found that the odds of being somewhat active (7500- 9999 steps/day) as opposed to being sedentary to low active (<7500 steps/day) required “good” self-rated health as well as ownership of a dog. Being active to highly active (> 10,000 steps/day) as opposed to sedentary to low active (<7500 steps/day), was related to participant’s city of residence, “good” self-reported health, not owning a vehicle, having a dog and being employed [47]. In another study [49] the data was dichotomised for ≥10,000 steps/day and the logistic regression was run using demographic and individual factors as explanatory variables for both males and females separately. From the analysis it was found that men who were less likely to achieve ≥ 10, 000 steps/day were more likely to be at least 60 years of age and overweight, while men who regularly walked in the workplace, who did vigorous activity at work or were employed in a blue collar occupation were more likely to achieve the above target.

Logistic regression models were also used to investigate whether the parent’s achievement of a daily step count goal, and non-excessive screen time on weekdays and weekends, were associated with children’s achievement of their daily step count goals [34, 50]. In Sigmundova’s study [50], the odds ratios were derived for meeting the recommended level of child step counts on weekdays and weekend days, separately for mother-child and father-child pairs, while controlling for the children’s and parent’s age and overweight/obese condition. It was found that on weekdays, the children are six times more likely to achieve their recommended level of step counts, where the daily kindergarten routine accounted for more than the median step count. Similarly, it was found that children are 10 times more likely to achieve the recommended level of step counts on weekends when their mothers achieve 10,000 steps per day or more.

### Interventions

Thirty six percent of the “effects *ON* step counts” studies have analysed effectiveness of an intervention using various statistical methods. Most of the studies (72%) out of 25 have used general linear models. It was noted that more than 50% of the studies using general linear models were analysing data for randomized control trials, followed by quasi-experimental, cross sectional and longitudinal designs. All the studies which used multilevel modeling involved randomized control trial designs.

It has been confirmed that well-structured pedometer walking programs with goals and proper physical activity consultations are very effective in promoting and changing the walking behavior in various communities [18, 52–54]. In one study [52] participants were randomly assigned to four groups, where group 1 and 2 had initial goals of 5000 steps per day and group 3 and 4 had initial goals of 10000 steps per day. Half-way through of the intervention groups number 2 and 4 were given new goals of 10000 steps per day and 5000 steps per day respectively. It was found that less walking behavior was observed when less ambitious goals were assigned regardless of whether the goals were met. However, a reduction in step goals also resulted in reduced walking behavior. It was found that pedometer-based physical activity interventions with counseling were effective for community-based as well as for work based individuals [13, 15, 16, 18]. These interventions promoted physical activity as well as reduced sitting time [16]. Similarly, it was found that creating awareness of the importance of physical activity within a pedometer based intervention for adolescents was also helpful in promoting physical activity [53]. The effectiveness of a competition based employer-sponsored physical activity program has been analysed using a one-way repeated measures analysis of variance with Duncan’s post hoc tests to examine in which program weeks significant changes occurred [55]. It was found that step counts tended to increase significantly in the middle of the 12 week program [55]. Apart from ANOVA, several studies have used analysis of covariance (ANCOVA) [4, 13, 13] and multivariate analysis of covariance (MANCOVA) analyses [4]. However, it should be noted that these models do not accomadate missing data [56]. If there is any missing data for an individual, then the entire data vector of that individual needs to be removed from the analysis [56]. Moreover, generally it is observed that the effectiveness and success of a physical activity program depends on the strategies and components included in the program. The long term effects of these strategies, helping participants to maintain levels of physical activity and health outcomes even after the program ends, are also very important. However, one study which examined a 4-year follow up effect for a 12 month pedometer based physical activity intervention found no support for this hypothesis, when using a repeated measures analysis of variance [17].

Apart from ANOVA, linear regression models have been used to examine the effect of environment on changes in pedometer recorded step counts during an intervention [20, 21]. An inverse association was found between density of private gyms and pedometer measured steps per day, such that those who live near higher density gym areas had a lower increase in step counts from baseline to six months [20] when controlling for potential confounders including age, race, education and sex. In the second study [21], the influence of the neighborhood environment on step counts during an intervention was assessed using hierarchical multiple linear regression models, while controlling for demographic variables. In the 12-month intervention one group was provided with a map of local areas to promote walking whereas the other group was only provided with pedometers to count their steps. Principal component analysis was used to reduce sixty-nine environment variables to 8 principal component factors, explaining 80.7% of the variability. The demographic variables were added in block 1 and 8 environmental factors including dangerousness of roads in block 2. Different environmental factors were found to be significant at different times (baseline, 3 months, 9 months and 12 months), however, none of the demographic variables (age, gender, annual household income and SIMD rank) were significant at any time [21]. This suggests that neighborhood environment is also important when promoting physical activity through an intervention.

Multilevel models were used to identify significant predictors for differences in step count changes for intervention and control groups [7, 30, 57, 58]. A feasibility study on classroom-based physical activity was analysed, adjusting for within cluster correlation at three different levels, namely student, classroom and school, using random effects for school, classroom and subject and assuming fixed effects for grade, gender, outdoor recess and physical education [7]. The effectiveness of goal manipulation along with social comparison feedback within an intervention have also been tested using multilevel models [57]. The results of this study agree with Anson’s study [52], confirming that there is an effect on physical activity behavior from social comparison feedback.

In two studies [30, 59] it was predicted that the percent of days on which step counts were logged would be higher during intervention periods than control periods, while controlling for age, physical activity level (life style index) and gender. The authors also used a multilevel model and a generalized estimating equation to predict the program’s participation [30]. These models have more flexibility in managing missing data as well as any unbalanced or nested structure in the data [56].

### Personal and psychological behavior

This Personal and Psychological Behavior category includes 14% of the “effect *ON* step counts” studies. All the studies which used structural equation modeling (SEM) fall in this category, with the rest using general linear models, generalized linear models and multi level models. In this category, most of the studies (80%) are cross sectional while only two studies that used structural equation models, have longitudinal designs. The four studies which used structural equation models relate to school-based activity programs [37, 60–62].

Structural equation modeling (SEM) has become a popular data-analytic approach among psychologists [63]. These models accommodate path analysis for measured variables [64] and latent constructs, reflected in their manifest indicators using measurement model [65]. The direct and indirect effects of variables such as self efficacy, social support, parental influence, environment, intrinsic and extrinsic goals and autonomous and controlled motivation on pedometer based physical activity have been tested [37, 60, 61]. In order to test the relationship between physical activity goals and autonomous controlled motivation and also to test the mediation effect of autonomous and controlled motivation for the relationship between goal content and physical activity, the sample was categorized on puberty status (physically immature vs physically mature), physical goal content (intrinsic goals vs extrinsic goals) and motivation for being physically active (autonomous motivation vs controlled motivation). It was found that there is no significant direct path from goal content to physical activity level. However, participant’s autonomous motivation acted as a mediator between goal content and physical activity level [37]. Similarly, SEM has been used for a three-wave prospective design which collected data at three different time stamps. This model was used to examine the positive effect of autonomous motivation towards exercise on predicted health-related quality of life, physical self-concept and the number of steps taken [62]. The model building process was comprised of two stages, a confirmatory factor analysis to test the measurement model followed by a path analysis [62]. Using path analysis, self efficacy was found to have direct and indirect effects on physical activity behavior, with parental influence affecting physical activity behavior directly for both parents and children [60]. However, SEM is also currently used for other important types of analysis such as longitudinal analysis, meta analysis, biometrical genetics, multivariate survival and spatial analysis, suggesting that more use of SEM methods is likely in the future [66].

A multivariate analysis of variance was used to estimate the main and interaction effects for gender, pubertal and weight status on goal content for physical activity behavior [37]. When considering general linear models, hierarchical linear regression was preferred by the researchers for testing the effects of planned behavior and other related psychological factors on physical activity [67, 68]. It was found that planned behavior predicts the intention to walk but not actual pedometer based step counts [68].

Finally, logistic regression was used to examine the effects of intention and actual television viewing on the physical activity of youths [35, 69]. Both these studies found that more hours watching television was associated with reduced step counts.

### Weather effects

Physical activity behavior is affected by the weather and seasonal patterns. The four studies which were categorized under this heading included a longitudinal study design, with two studies utilizing analysis of variance and the other two studies using a linear mixed modeling technique to analyse the data. In order to test the overall effect of seasons on step counts along with sociodemographic variables, ANOVA was used [70, 71]. It was found that, both normal and overweight participants living in the UK reduced their step counts in winter, with normal weight individuals more influenced by this seasonal change [70]. Furthermore, a significant day of the week effect (especially high for Sunday) existed in both summer and winter periods of the year [70, 71]. These studies considered daily weather variables for temperature, rainfall, relative humidity, wind speed, sea level pressure, snow on ground etc. along with demographic variables [72, 73].

The other studies found using multilevel models that the effect of temperature and humidity were both significant positive determinants of step counts while rainfall, snow depth and wind speed had a significant negative relationship with daily step counts [72, 73]. A significant interaction effect for maximum wind speed and BMI was also found, with maximum wind speed having more of an effect on individuals with lower (BMI = 20) and higher BMI’s (BMI = 35) [73]. Clearly, all these results suggested that the effect of weather and climatic conditions need to be considered when promoting physical activity interventions.

### Health

24% of all the papers were categorized as health related studies. This category can be further divided into “effect *ON* step counts” and “effect *OF* step counts”.

When considering the “effect *ON* step counts” one longitudinal study used multilevel modeling [74] to analyse the data. This study predicted the change in physical activity of weight conscious college women. For this analysis body satisfaction and reported eating behavior measures were considered as predictors in two models, one to examine the retrospective and the other the prospective relationship between physical activity and body/eating behavior. It was found that weight conscious college women tend to show a increase in physical activity level after a negative weight experience or negative eating behavior.

All the other health related studies were categorized under “effect *OF* step counts”. Only general linear models (52%) and generalized linear models (48%) were used in these studies. Most of the studies were cross sectional in design with two longitudinal and one non randomized controlled trial. Linear regression and ANOVA general linear models were used. Logistic regression was the most common (80%) generalized linear model approach, followed by exponential regression and log-binomial regression.

Logistic regression has been extensively used to predict the presence or absence of health related outcomes such as adiposity measures [28, 75], coronary heart disease [36, 76–78], bone and muscle health [27, 29], based on physical activity levels. In all these studies physical activity was measured by step counts and used as a predictor along with other relevant independent variables. In ANOVA analyses, step counts were divided into tertiles [36, 79–81], whereas in linear regression, correlations with step counts were considered [19, 27–29, 82]. Seniors who walked more, maintaining the recommended level of physical activity, have less risk of sarcopenia [27, 29]. Also more steps per day lowered the odds of having Metabolic Syndrome (MetS) [36, 77, 78]. Log binomial is another modeling method that can be utilized depending on the outcome variable and objectives of the study. In these models, a log function is used as the link function instead of a logit function. Log binomial regression is able to produce an unbiased estimate for the adjusted relative risk [83]. These models can be used to derive the risk ratios instead of odds ratios when the outcome event is rare. One study used the log binomial regression to investigate the association between physical activity (steps/day) and depression [84]. According to this study, women with moderate ambulatory levels (>7500 steps/day) had a 50% lower prevalence of depression compared with sedentary women (<5000 steps/day). Hierarchical linear regression was also used to evaluate the relationship between cognition and physical activity [85]. In this study cognitive performance was measured by several scores including verbal episodic memory and visual episodic memory etc.

### Other- Miscellaneous

Only eight articles (10%) were categorized in this Other-Miscellaneous category. Most of these studies had a cross sectional design, with some longitudinal and randomized control trials. All these articles used ANOVA and linear regression.

Based on physical activity recommendations [81, 86], linear regression was used to relate step counts to 30 minutes of moderate to vigorous physical activity level per day.

Similarly maximum oxygen usage during high intensity exercise was predicted using a linear regression model based on step counts and other related body composition measures [87]. Other studies examined the influence of monitoring interval and alternative starting days for step count interventions in order to reliably assess and predict the participants’ physical activity [31, 88]. ANOVA, inter class correlation and linear regression methods were used to model the collected data. One study reported that a minimum of four days need to be monitored starting from Sunday [31]. Another study [88] also claimed that four days were required for a pedometer based intervention, with different monitoring periods for different step count collecting devices.

## Discussion

In this review we identified four main statistical approaches for describing step counts obtained through physical activity programs; namely general linear models, generalized linear models, multilevel models and structural equation models. The most popular method of analysis is analysis of variance followed by linear regression and generalized linear models, including logistic regression, exponential regression and log binomial regression. Multilevel models are the third most preferred method followed by structural equation models. According to the studies in this review, most of the studies are observational where the researchers collected “free living” pedometer steps from the participants. Cross sectional study designs are the most common observational study design followed by longitudinal studies and case control studies. When considering experimental study designs, randomized control trials are most popular followed by quasi experimental designs and non-randomized control trials.

Most of the studies with a cross-sectional design have been analysed with a combination of general linear models and generalized linear models, structural equation modeling and multilevel modeling. Studies with longitudinal designs have commonly used general linear models, however, multilevel models and structural equation models have also been used to examine step count effects. Most of the randomized control trials have utilized general linear models or multilevel models in their analysis.

When considering the “effect *ON* step counts”, the studies used step counts either as a continuous variable or step count ranges (categorical). When considering the generalized linear regression, the majority of the studies used logistic regression, categorizing the step counts as binary outputs (eg:- attainment of 10,000 steps or not). However, ordinal logistic regression was also used when the step counts were divided into more than two categories. When considering the “effect *OF* step counts” on health outcomes, for example the effect of step counts on adiposity measures (eg:- body fat percentage, BMI) [28], with longitudinal study designs, generalized linear models and general linear models have been preferred using mean step counts per day as a predictor [28].

Structural Equation Models (SEM) examined the direct, indirect and mediation effects of psychometric properties such as self-efficacy and autonomous/controlled motivation on step counts. In these studies they also tested the effect of psychosocial models such as social cognitive theory on physical activity [60, 61]. However, several studies stressed the need for large sample sizes in order to obtain reliable covariance estimates for SEM measurement models [60, 62]. These models were also used to study the physical activity behavior of children. Parceling techniques have been used to reduce the complexity of these measurement models when sample sizes are smaller, while also improving the normality of the variables used in the models [62]. Missing data imputation can be conducted within a structural equation modeling framework [64]. It is noted that, the total variance explained using these models can be improved through the consideration of other effects such as environmental and sociodemographic factors. There are many more ways to explore the impacts of physical activity programs using structural equation modeling, in areas such as latent class growth models for longitudinal data, multilevel analysis, meta analysis and multi-group analysis, in order to discover causal structures within a SEM framework [89].

Multilevel models are capable of providing a correction for the error structure for repeated measurements for the same individual over time [72, 73]. These models do not assume equal numbers of repeated observations for each participant, ensuring that the inclusion of respondents with missing data does not bias estimated effects [74, 90]. It was found through gender stratified bivariate and multivariate random effect models that neighborhood safety at night is an important factor for predicting the physical activity levels of women [46]. Similar studies suggested that goal setting for physical activity were effective for some participants [58]. Importantly autocorrelation in repeated measures step count data must be addressed in analysing randomized control trials, as it may lead to cumulative carry over effects, especially when there are linear time trends [58].

Factors relating to family, demographics, community and weather have been identified, suggesting that physical activity policies and interventions need seasonal and socio-demographic adaption, in order to motivate participants to be more active [34, 38, 43, 70]. Weight consciousness has also been associated with the response of individuals’ engagement in an activity program, over time. However this information has rarely used for the personalisation of the physical activity programs. When analysing longitudinal data, there often exist complex error structures and missing values [56], making the use of multi-level models necessary.

## Limitations

There are some limitations for this systematic review. In this systematic review, we only considered studies which contained a study variable related to step counts, collected from a healthy sample using a pedometer or some such device. Any article which collected step count data through physical activity questionnaires has therefore been omitted. In addition, the review was limited to peer reviewed articles published in English and only to articles that performed a quantitative analysis using a statistical modeling technique. Finally, a meta-analysis has not been performed due to the wide range of statistical methods applied, the wide range of research objectives and populations.

## Conclusions

In summary, this systematic review was able to identify four main classes of statistical modeling methods that have been used to analyse step count data obtained through physical activity programs. They are: general linear models, generalized linear models, multilevel models and structural equation models. It has been found that step count behavior depends on psychosocial, demographic, weather and climatic factors. Therefore, these factors should be controlled when using step counts to assess program outcomes. However, none of the studies in the review have used time series modeling methods to analyse step count data. These methods can better account for the serial autocorrelation in long step count series, allowing unbiased estimation.

It is important that individuals who enroll in a physical activity program are appropriately motivated along their journey if they are to achieve successful outcomes. This can be achieved by detecting changes in physical activity levels using change-point detection methods with daily step counts. This could allow more personalised program development and hence successful program outcomes. Similarly the review identified no use of machine learning and text mining approaches for acquiring a better understanding program motivation. For this reason it is recommended that future studies should utilize time series modeling (univariate and multivariate), change-point analysis methods, machine learning techniques such as cluster analysis, random forest etc., and text mining approaches to better understand the factors underlying step count patterns and program involvement.

In addition it is recommended that intervention combinations should be trialled in order to enhance the personalisation of physical activity programs. Furthermore, the effect of individualized step by step goal setting on long term physical activity behavior should also be tested in future studies.

## Supporting information

S1 File PRISMA checklist. (DOC)

S1 Table Characteristics of studies. (DOCX)

S2 Table Summarized table. (XLSX)
