# Supplementary material for: Statistical methods to model and evaluate physical activity programs, using step counts: A systematic review
Source: PLoS One. 2018 Nov 2;13(11):e0206763. doi: 10.1371/journal.pone.0206763 (PMC6214537; doi:10.1371/journal.pone.0206763)
Supplement: S1 Table — (DOCX) [file pone.0206763.s002.docx]

| **Author** | **Study Design** | **Sample** | **Country of the sample** | **Analysis Desciption** | **Outcome Measure** | **Statistical Model** | **Scope** |
| --- | --- | --- | --- | --- | --- | --- | --- |
| Anson, Denis and Madras, Diane [22] | Randomized Controlled Trial | N=80 (Females= 69, Males= 11), Mean Age= 24 years, N Complete= 53 | United States of America | 4 × 2 analysis of variance with interaction effects goal assign intervention | Steps logged per day | General Linear Models \| ANOVA | Intervention |
| Baker, Graham, Gray, Stuart R., Wright, Annemarie, Fitzsimons, Claire, Nimmo, Myra, Lowry, Ruth and Mutrie, Nanette [21] | Randomized Controlled Trial | N= 79, (Females= 63, Males= 16), Mean Age= 42.9 (±8.9) | Scotland | Steps/day and health related outcome data were analyzed using two-way mixed factorial analyses of variance (ANOVA) | Steps per Day | General Linear Models \| ANOVA | Intervention |
| Behrens, Timothy K., Domina, Lome and Fletcher, Gena M. [23] | Cross-sectional study | N=640 | United States of America | Examined whether steps per week for each group were significantly different by each week of the program | Steps per Day | General Linear Models \| ANOVA | Intervention |
| De Cocker, Katrien A., De Bourdeaudhuij, Ilse M., Brown, Wendy J. and Cardon, Greet M. [77] | Randomized Controlled Trial | N= 866 (aged 25 to 75), From Ghent= 440 (Mean age: 49.8±13.1, Men: 47.5%, Employed: 62.4%), From Aalst= 426 (Mean age: 50.0±12.9, Men: 52.6%; Employed: 54.4%) | Belgium | Evaluated the effect of the campaign on step counts and sitting time, with time (baseline/follow-up) as the within participants factor, community (intervention/comparison) as the between participants factor, and age and number of years education as covariates. | Steps per Day | General Linear Models \| ANOVA | Intervention |
| De Cocker, Katrien A., De Bourdeaudhuij, Ilse M., Brown, Wendy J. and Cardon, Greet M. [24] | Cross-sectional study | N (Intervention) = 216 and N (Comparison)= 204 | Belgium | Evaluated the long-term effects of the intervention on pedometer-based and self-reported physical activity, with time (baseline 2005 and follow-up 2009) as within-subjects factor and community (intervention and comparison community) as between-subjects factor. | Steps per Day | General Linear Models \| ANOVA | Intervention |
| Chia, M. [73] | Cross-sectional study | N=900, Primary pupils (aged 9–12 years)=312,Secondary pupils= 288 (aged 13–16 years), and Junior College Students= 300 (aged 17– 18 years) | Singapore | The data set segregated by sex and schooling levels to analyze aggregated step count for (i) within school and outside school and (ii) for the weekday and weekend day. One-way ANOVA was used to examine (iii) sex differences in mean step count and (iv) step count across the schooling levels. | Aggregated Step Counts | General Linear Models \| ANOVA | Family, Community and Sociodemographic |
| Clemes, S. A., Patel, R., Mahon, C. and Griffiths, P. L. [75] | Cross-sectional study | N= 72, Female= 60%, Mean age 37 (±13), Mean BMI 24.0 (±3.5) kg/m2 | United Kingdom | The study compared group differences and total step counts reported on each day of the week using repeated measures ANOVA, with Bonferroni-corrected post hoc comparisons. | Mean Step Counts per day and Mean Sitting Time per day | General Linear Models \| ANOVA | Family, Community and Sociodemographic |
| Croteau, K., Schofield, G., Towle, G. and Suresh, V. [76] | Cross-sectional study | N= 72, Girls= 43 (60%), Boys= 29, Mean age = 9.8 ± 1.1, Age range = 8–12 years, Body Mass Index (BMI) (kg/m2)= 15.8 ±1.9 | Kenya | Data analysis included descriptive statistics and 2-way ANOVA (age x gender) | Steps per day for the measuring period | General Linear Models \| ANOVA | Family, Community and Sociodemographic |
| Dlugonski, D. and Motl, R. W. [78] | Cross-sectional study | N = 66 , Unmarried mothers = 22 (Mean BMI= 27.18 (6.47)); Married mothers = 22 (Mean BMI= 26.12 (5.95)); Non-mothers = 22 (11 married, 11 unmarried)(Mean BMI= 26.74 (5.98) ) | United States of America | Tested for initial differences in descriptive characteristics among groups. ANCOVA was used to identify overall differences in physical activity among the three groups while adjusting for covariates | Objectively measured Steps per Day | General Linear Models \| ANCOVA | Family, Community and Sociodemographic |
| Payn, T., Pfeiffer, K. A., Hutto, B., Vena, J. E., LaMonte, M. J., Blair, S. N. and Hooker, S. P. [15] | Cross-sectional study | N=85, Male=70.6%, Mean age = 57.9 years (±10.9) | Columbia | Examined differences in daily steps between categories of self-rated health, BMI, meeting the PA recommendation, gender, race, and age | average Steps/day | General Linear Models \| ANOVA | Family, Community and Sociodemographic |
| Pillay, J. D., Kolbe-Alexander, T. L., van Mechelen, W. and Lambert, E. V. [86] | Cross-sectional study | N=70, Male=35, Female=35, Mean Age=32 (± 8)yrs | South Africa | ANOVA, adjusting for age, gender were used to compare groups namely LOW (< 5000 steps/d, irrespective of intensity), HIGH-LOW (> 5000 steps/d with no aerobic activity) and HIGH-HIGH (> 5000 steps/d with aerobic activity) | Volume and intensities of daily steps- LOW (< 5000 steps/d, irrespective of intensity), HIGH-LOW (> 5000 steps/d with no aerobic activity) and HIGH-HIGH (> 5000 steps/d with aerobic activity) | General Linear Models \| ANOVA | Health Related |
| Chan, C. B., Spangler, E., Valcour, J. and Tudor-Locke, C. [71] | Cross-sectional study | N=179, Female (86.8%), Mean Age= 43, Mean BMI= 29 | Canada | Examined the cross-sectional relationship of an objective measure of walking (pedometer-determined steps/ day) and general indicators of health (i.e., BMI, waist circumference, resting heart rate, and blood pressure) and a previous diagnosis of one or more components of the metabolic syndrome.-BMI | At lest one factor other than obesity -metabolic syndrome And BMI as normal weight, overweight, or obese | General Linear Models \| ANOVA | Health Related |
| Newton, R. L., Jr., Han, H., Johnson, W. D., Hickson, D. A., Church, T. S., Taylor, H. A., Tudor-Locke, C. and Dubbert, P. M. [18] | Cross-sectional study | 379 African American adults (mean age 60.1 years; 60% female) | United States of America | Assessed significant differences in demographic characteristics between participants across tertiles (<3717 steps/day, 3717–6238 steps/day, >6238 steps/day) | steps/day -tertiles | General Linear Models \| ANOVA | Health Related |
| Clemes, Stacy A., Hamilton, Sarah L. and Lindley, Martin R. [74] | Cross-sectional study | N= 86 (normal-weight -BMI <25 kg/m2, Mean age = 34 ± 12.1 years); N= 91 (overweight BMI= 25–29.9 kg/m2, Mean age = 40.6 ± 13.6 years) and N= 75 (obese- BMI >30 kg/m2, Mean age = 41.2 ± 12.4 years) | United Kingdom | Tested whether mean step counts differed between days. Differences in mean step counts and demographic variables between the three groups were tested using one-way ANOVAs, with Bonferroni post hoc comparisons | Mean Step Counts per day And Activity patterns (Steps per Day) over different days of the week | General Linear Models \| ANOVA | Health Related |
| Clemes, Stacy A., Hamilton, Sarah L. and Griffiths, Paula L. [65] | Within-subjects repeated-measures design | N= 96; N= 45 (normal-weight) (Female= 58%, Mean age = 39.1 (± 12.4) years, Mean BMI = 22.2 (± 2.1) kg/m2) and N= 51 (overweight), Female= 49%, Mean age = 42.1 (± 12.5) years, Mean BMI = 29.3 (± 4.5) kg/m2) | United Kingdom | Tested for an overall effect of season, and for an interaction between seasonal change in steps and weight status, a 2-way mixed ANOVA was conducted with season (summer and winter mean steps) as the within-subjects factor and weight status as the between subjects factor (normal weight versus overweight). | Steps per Day | General Linear Models \| ANOVA | Weather and demographic |
| Craig, C. L., Tudor-Locke, C., Cragg, S. and Cameron, C. [25] | Cross-sectional study | Boys=5823, Girls= 5581, Age Range= 5- to 19-yr-olds | Canada | Reactivity was assessed by examining estimates from the pattern of pedometer data across days (arranged from first day of collection to last) using a repeated-measures ANOVA. | Steps per Day | General Linear Models \| ANOVA | Other |
| Fitzsimons, C. F., Baker, G., Gray, S. R., Nimmo, M. A. and Mutrie, N. [79] | Randomized Controlled Trial | N= 79 (100%) [Group 1: 39 (49%), Female= 79 (31%), Mean Age= 47.3 (9.3) and Group 2: 40 (51%)), Female= 80 (32%), Mean Age=51.2 (7.9) | United Kingdom | Analyses were performed on an intention to treat basis (baseline value carried forward for missing data) using mixed-factorial ANOVAs and follow-up t-tests. | Steps per Day during the pre intervention and post intervention | General Linear Models \| ANOVA | Intervention |
| Gilson, Nicholas, McKenna, Jim, Cooke, Carlton and Brown, Wendy [80] | Randomized Controlled Trial | Female= 58, Mean age= 42 (±10) years and Male= 6, Mean age= 40 (±11) years | United Kingdom | To identify significant differences for (a) preintervention data and (b) intervention effects. | Steps per Day during pre and intervention time | General Linear Models \| ANOVA | Intervention |
| Gilson, Nicholas D., Puig-Ribera, Anna, McKenna, Jim, Brown, Wendy J., Burton, Nicola W. and Cooke, Carlton B. [81] | Randomized Controlled Trial | N (White collar university employees)= 179; Mean age= 41.3 (± 10.1) years, Women= 141; N(UK) = 64; Mean age = 41.4 ± 10.4 years, Women= 58; N (Australia) = 70; Mean age = 43.1 ± 10.8 years, Women= 54) and N (Spain)= 80; Mean age= 39.1 ± 9.7 years, Women= 58 | United Kingdom, Australia, Spain | Compared Workday step counts and reported sitting times for pre-intervention and intervention measurement | Work day Mean step counts at the beginning, mid and end of the intervention | General Linear Models \| ANOVA | Intervention |
| Hamilton, S. L., Clemes, S. A. and Griffiths, P. L. [83] | Within-subjects repeated-measures design | NN= 96, Male= 52%, Mean age= 41.0 (12.3) years, Mean BMI= 26.1 (5.1) kgm–2 | United Kingdom | ANOVA (with Greenhouse–Geisser correction applied) was employed to assess whether step counts varied according to the day of the week within each season. | Summer steps per day And Winter steps per day | General Linear Models \| ANOVA | Weather and demographic |
| Lubans, D. R., Morgan, P. J., Callister, R. and Collins, C. E. [85] | Randomized Controlled Trial | N = 124, Mean age= 14.1 (6 .8) years | Australia | Objectively recorded physical activity (mean steps/day), self-reported sedentary behavior, and dietary habits were measured at baseline and at 6-month follow-up and intervention effects were assessed. | Effect of the intervention (From baseline to follow up) on steps per day | General Linear Models \| ANOVA | Intervention |
| Pelclová, Jana, Gába, Aleš, Tlučáková, Lenka and Pośpiech, Dariusz [7] | Cross-sectional study | N=167, Mean age= 62.8 (4.8) years; N (Czech Republic)= 45, Mean age= 64.2 (3.8), Mean BMI= 26.1 (3.6), N (Slovakia)= 51, Mean Age= 61.8 (5.0), Mean BMI= 27.5 (4.2), N (Poland)= 71, Mean Age= 62.5 (5.2), Mean BMI= 27.9 (4.5) | Czech Republic, Slovakia, Poland | Tested whether body composition variables (dependent variables: BMI, BFMI, FFMI, WHR, VFA) were associated with achievement of different PA guidelines (independent variables): <7500 steps/day, 7500–9999 steps/day, 10,000–12,500 steps/day, >12,500 steps/day) | Effect of body composition variables in steps per day | General Linear Models \| ANOVA | Health Related |
| Puig-Ribera, A., McKenna, J., Gilson, N. and Brown, W. J. [87] | Randomized Controlled Trial | N= 70 ; Men= 21, BMI= 25.09 (± 2.26) kg/m2, waistcircumference= 88.7 (± 8 cm) and Women= 49, BMI = 23.63 (±3.82)kg/m2, waist circumference = 79 (± 10.4 cm). | Spain | One-way ANOVA was used to examine differences between groups. | Work day Step Counts | General Linear Models \| ANOVA | Other |
| Raustorp, Anders, Pagels, Peter, Fröberg, Andreas and Boldemann, Cecilia [89] | Cross-sectional study | 105 second-grade pupils (42 girls) from two city schools and 105 (52 girls) fifth-grade pupils from all five schools. | Sweden | One-way ANOVA Scheffe post hoc was used to analyse the difference between the three second-grade cohorts in 2000, 2006 and 2013. | Change in Physical Activity over time- Steps per Day | General Linear Models \| ANOVA | Family, Community and Sociodemographic |
| Samuels, Tiana Y., Raedeke, Thomas D., Mahar, Matthew T., Karvinen, Kristina H. and DuBose, Katrina D. [88] | Randomized Controlled Trial | N= 43 (faculty and staff volunteers from a large university), Female= 35, Mean Age= 48.8 (9.1), **BMI-pre (kg.m−2)= 29.1 (5.1)** | United States of America | Repeated measures ANOVA revealed that the 10 K group showed the largest increase in step counts whereas the bouts group showed the smallest change over the intervention period | average Steps/day each week | General Linear Models \| ANOVA | Intervention |
| Sigmundova, D., Vasickova, J., Stelzer, J. and Repka, E. [12] | Longitudinal Study | Eligible subjects (641) were selected from a sample of 906 university students | Czech Republic | To determine the most effective starting day | Mean steps per day for each day of the week | General Linear Models \| ANOVA | Other |
| Brusseau, T. A., Burns, R. D. and Fu, Y. [13] | Prospective and observational research design | N= 232, boys=144 (62%), Mean age= 13.3 (± 0.4) years. | United States of America | To determine the effect size and practical significance of each pair-wise comparison. | pedometer step counts per minute | General Linear Models \| ANCOVA | Intervention |
| De Cocker, Katrien A., De Bourdeaudhuij, Ilse M. and Cardon, Greet M. [19] | Quasi-experimental controlled pretest–posttest design | Intervention group (Pre= 146, Mean Age=39.9 (± 9.8), Men = 42.2%; Post= 68, Mean Age= 41.4 (± 9.6), Men= 50%), Comparison Group (Pre= 152, Mean Age= 37.5 (± 8.7), Men= 52.1%; Post= 79, Mean Age= 37.8 (± 8.9), Men= 43.4%) | Belgium | Analyzed the effect of the intervention on pedometer-based and self-reported PA repeated measures analysis of covariance was conducted with time (baseline/ follow-up) as the within-subjects factor, and worksite (intervention/comparison) and risk profile (<10 000 steps/day at baseline = at-risk group vs >10 000 steps/day at baseline) as between subjects factors. | Daily pedometer step counts (steps/day) | General Linear Models \| ANCOVA | Intervention |
|  |  |  |  | Evaluated the effect of the intervention on workday and non workday step counts. | Non-workday pedometer step counts (steps/non-workday) | General Linear Models \| ANCOVA | Intervention |
| Yasunaga, Akitomo, Togo, Fumiharu, Watanabe, Eiji, Park, Hyuntae, Park, Sungjin, Shephard, Roy J. and Aoyagi, Yukitoshi [66] | Longitudinal Study | Male=41, Mean Age =71.2 (± 0.7) Mean Height= 1.61 (± 0.01)m,Body Mass= 59.4 (± 1.3), Body-mass index (BMI) 23.0 (± 0.5) and Female=54 female, Mean Age=71.6 (±0.6), Height=1.48 (± 0.01) m, Body Mass=51.8 (± 0.8) kg , BMI=23.6 (± 0.3) kg/m2), Age range=65–83 yr | Japan | ANCOVA assessed independent associations between year-averaged physical activity variables and sex (after controlling for age) and age group (65–74 vs. 75–83 years) after controlling for sex | Year-Averaged daily step count | General Linear Models \| ANCOVA | Family, Community and Sociodemographic |
| Brusseau, T. A., Burns, R. D. and Fu, Y. [13] | A prospective and observational research design | N= 232, boys=144 (62%), Mean age= 13.3 (± 0.4) years. | United States of America | Examined the effect of activity mode (motor skills and games, fitness), environment (indoors, outdoors), and semester (Fall, Spring) on pedometer step counts per minute and estimated time in MVPA, controlling for the clustering of daily physical activity measurements within each student and the clustering of students within classrooms. | pedometer step counts per minute during the physical education duration | General Linear Models \| MANCOVA | Intervention |
| Hart, T. L., Swartz, A. M., Cashin, S. E. and Strath, S. J. [84] | Cross-sectional study | N=52, Mean age = 69.3 (±7.4) years, Age range= 55-86 years | United States of America | Determined between day differences in mean PA level for each intensity | Steps per day | General Linear Models \| RMANOVA | Other |
| Seghers, J., Vissers, N., Rutten, C., Decroos, S. and Boen, F. [20] | Cross-sectional study | N= 1272 (Boys= 547 and Girls= 725), Mean age= 11.42 years (±0.47). | Belgium | A multivariate 2 X 2 X 2 ANOVA was used to examine the main and interactions effects of gender (boy vs. girl), weight status (normal weight vs. overweight) and pubertal status (physically immature vs. physically mature) on goal content for leisure-time sport and PA (intrinsic goals vs extrinsic goals), behavioural regulation (Autonomous motivation vs Controlled motivation) and average steps per day. | Motivational and Behavioural outcomes with Physical activity (objectively meausred average step count per day) | General Linear Models \| MANOVA | Personal and psychological behaviour |
| Boyer, K. A., Kiratli, B. J., Andriacchi, T. P. and Beaupre, G. S. [69] | Cross-sectional study | N=105, Females=66 (63%), Mean Age (Female)=56.5 (±4.2) | United States of America | To investigate the relationship between Bone Density Index and Bone Mineral Density, steps/leg/day and BMD and walking speed and BMD for females and males | Femoral bone mineral density (BMD)- For Tscore=(-1) | General Linear Models \| Linear Regression | Health Related |
| Cao, Z. B., Miyatake, N., Higuchi, M., Miyachi, M., Ishikawa-Takata, K. and Tabata, I. [70] | Cross-sectional study | N=940 Japanese adults, Female=480 (51%), Mean Age= 44.7 (±13.9), Mean BMI (kg/m2)=22.0 (±2.9) | Japan | To estimate relationships between step count per day and MVPA and PAEE | Steps per day recommendation | General Linear Models \| Linear Regression | Health Related |
| Cao, Zhen-Bo, Oh, Taewoong, Miyatake, Nobuyuki, Tsushita, Kazuyo, Higuchi, Mitsuru and Tabata, Izumi [34] | Cross-sectional study | N=940 Japanese adults, Female=480 (51%), Mean Age= 44.7 (±13.9), Mean BMI (kg/m2)=22.0 (±2.9) | Japan | To estimate relationships between step count per day and MVPA and PAEE | Steps per day recommendation | General Linear Models \| Linear Regression | Health Related |
| Park, H., Park, S., Shephard, R. J. and Aoyagi, Y. [8] | Cross-sectional study | N=175 (Women=55%), Mean Age (years)=72.5 (±4.6), Mean Body mass index= (kg/m2)=23.6 (±3.0) | Japan | Tested associations between habitual physical activity and muscle mass. ANCOVA assessed independent associations between the four categories of physical activity and muscle mass after controlling for age and/or sex. | Degree of sarcopenia (age-related condition characterized by loss of muscle mass, with a concomitant decline of voluntary muscle strength and increase of fatigability ) | General Linear Models \| Linear Regression | Health Related |
| Thomson, J. L., Landry, A. S., Zoellner, J. M., Connell, C., Madson, M. B., Molaison, E. F. and Yadrick, K. [90] | Non randomized uncontorlled trial | N=269, African American (94%), Female (85%), overweight or obese (91%), Mean Age= 44 years(± 12.2) | United States of America | Determined the predictive ability of the adherence indicators (including education session attendance (ESA) And weekly steps/day pedometer diary submission (PDS)) for health outcome changes in the presence of covariates. | % Body fat, Fat mass, Low-density lipoprotein, Diastolic blood pressure And Steps per day | General Linear Models \| Linear Regression | Health Related |
| Tudor-Locke, C., Schuna, J. M., Han, H., Aguiar, E. J., Green, M. A., Busa, M. A., Larrivee, S. and Johnson, W. D. [26] | Cross-sectional study | N= 3388, Men= 1725 (51%), Mean Age= 46.8 years, Mean BMI= 28 | United States of America | Evaluated the independent associations of steps per day, peak 30-min cadence, percent of time at zero cadence with a subset of evaluated cardiometabolic variables. | BMI (kgm-2), SBP (mm Hg), Glucose (mgdL-1), Insulin (KUImLj1), HDL cholesterol, mgIdLj1, Triglyceride, mgIdLj1 And Glycohemoglobin, % | General Linear Models \| Multiple Linear Regression | Health Related |
| Park, H., Togo, F., Watanabe, E., Yasunaga, A., Park, S., Shephard, R. J. and Aoyagi, Y. [10] | Cross-sectional study | N=172, (Male=76, Female=96), Mean Age (Men)=73.0 (±4.4) | Japan | Associations between bone health and the quantity and quality of habitual physical activity | T-score of calcaneal osteosonic index -Men | General Linear Models \| Linear Regression | Health Related |
| Craig, C. L., Cameron, C. and Tudor-Locke, C. [16] | Longitudinal Study | N=539 (out of 1,187 eligible children (Age Range= 5–19 years)), Boys=256 (47%, Mean age= 12.3 years) and Girls=283 (53% , Mean age=11.4 years) | Canada | Associations between steps/day for parents and children (controlling for their age and sex) were estimated using general linear and logistic regression. | Steps/day-Son | General Linear Models \| Linear Regression | Family, Community and Sociodemographic |
| Hajna, S., Ross, N. A., Joseph, L., Harper, S. and Dasgupta, K. [82] | Cross-sectional study | n=2949, Mean Age= 46.6 (±16.4)years, Mean BMI = 27.3 (±5.5) kgm2 | Atlantic Canada, Québec, Ontario, the Prairies and British Columbia. | Linear regression models were used to estimate mean differences in steps/day across quartiles of walkability.-GIS derived walkability | accelerometer-assessed daily steps. | General Linear Models \| Linear Regression | Family, Community and Sociodemographic |
| Kari, J. T., Pehkonen, J., Hirvensalo, M., Yang, X. L., Hutri-Kahonen, N., Raitakari, O. T. and Tammelin, T. H. [28] | Longitudinal Study | N=753 (64% women) , Mean Age= 41.7 years | Finland | Evaluated the associations between income and physical activity. | Physical Activity which interms of steps per day or aerobic steps per day | General Linear Models \| Linear Regression | Family, Community and Sociodemographic |
| Matthiessen, Jeppe, Andersen, Elisabeth Wreford, Raustorp, Anders, Knudsen, Vibeke Kildegaard and Sørensen, Mette Rosenlund [14] | Cross-sectional study | N=205, Mean Age=45.2 (±14.6) (2007–2008); n=1419 (2011–2012) Mean Age= (47.2 (±15.6)) | Denmark | Continuous outcomes, i.e. steps/day, cadence and steps/day incl. cycling, were analysed using linear regression models; | pedometer measured PA (steps/day, cadence, step-defined activity levels, and steps/day incl. cycling) | General Linear Models \| Linear Regression | Family, Community and Sociodemographic |
| Vander Ploeg, K. A., Kuhle, S., Maximova, K., McGavock, J., Wu, B. and Veugelers, P. J. [32] | Cross-sectional study | N=1355, Girls= 717 (52.9%), Mean Age= 10.9, Mean BMI= 19.4 | Canada | Determined the associations with girls’ and boys’ step-counts taken on weekend days and on school days. Secondly multivariable linear regression models to adjust for the confounding potential of parental educational attainment, household income, and year of data collection (referred to as Model 1). Last, the study considered parent beliefs and support variables simultaneously while adjusting for the above confounders to quantify their independent importance for children’s step-counts (referred to as Model 2). | Daily pedometer step counts | General Linear Models \| Linear Regression | Family, Community and Sociodemographic |
| Burns, R. D., Brusseau, T. A. and Hannon, J. C. [33] | Interrupted Time Series Design (ITSD) quasi-experimental framework | N=327 students (Girls = 162, Boys = 165) from the fourth and fifth grades, Mean age= 9.60 (± 1.75) years | United States of America | Piecewise regression examined pre- and postintervention intercepts and slopes, and the change in these parameters using postestimation statistics. | Change in School Day step count | General Linear Models \| Linear Regression | Intervention |
| Pitts, S. B. J., Keyserling, T. C., Johnston, L. F., Evenson, K. R., McGuirt, J. T., Gizlice, Z., Whitt, O. R. and Ammerman, A. S. [30] | Randomized Trial | N=249, Mean age=56.5 years, Mean BMI (calculated as kg/m2)= 36.3 | United States of America | Examined the associations between the change variables and food and PA environments in order to assess moderation. | Change in pedometer-measured steps | General Linear Models \| Linear Regression | Intervention |
| Tudor-Locke, C., Leonardi, C., Johnson, W. D., Katzmarzyk, P. T. and Church, T. S. [35] | Cross-sectional study | N=3523, Males=1781 (51%) and Females=1742, Age range=20– 85 years of age | United States of America | Predicted steps/day (summed over the week) corresponding to minimally 150 min/week of MVPA. | steps per day | General Linear Models \| Linear Regression | Other |
| Sigmundova, D., Vasickova, J., Stelzer, J. and Repka, E. [12] |  | Eligible subjects (641) were selected from a sample of 906 university students | Czech Republic | Determined the most effective monitoring interval for step counts intervention | Mean steps per day for each day of the week | General Linear Models \| Hierarchical regression | Other |
| Robertson, L. B., Ward Thompson, C., Aspinall, P., Millington, C., McAdam, C. and Mutrie, N. [31] | Randomized Controlled Trial | n = 79, Male=16 (20%), Mean age= 51 years | Scotland | Relationship between environment factors and (i) step counts, and (ii) the change in step counts relative to baseline, was examined using hierarchical multiple linear regression, controlling for age, gender, income, and deprivation. | Influence of the local neighbourhood environment on steps/day-(baseline, 3 months, 6 months and 12 months) | General Linear Models \| Hierarchical Multiple Linear Regression | Intervention |
| Hayes, Scott M., Alosco, Michael L., Hayes, Jasmeet P., Cadden, Margaret, Peterson, Kristina M., Allsup, Kelly, Forman, Daniel E., Sperling, Reisa A. and Verfaellie, Mieke [27] | Cross-sectional study | N (Young) = 29, Mean Age= 21.2 (±3.2), Mean BMI= 22.8 (±2.9) and N (older adults) = 31, Mean Age= 64.5 (±7.0), Mean BMI= 25.6 (±4.5) | canada | Evaluated relationships between cognition and physical activity | Cognitive performance measured by executive function composite score, verbal episodic memory composite score, visual episodic memory composite score, and face-name accuracy | General Linear Models \| Hierarchical linear regression | Health Related |
| Lubans, David Revalds and Morgan, Philip James [29] | Cross-sectional study | N=119, Age Range =14—15 year old students from three secondary schools | Australia | Analyzed the relationship between potential correlate factors and physical activity (defined as mean steps/day). | steps/day | General Linear Models \| Hierarchical linear regression | Personal and psychological behaviour |
| Scott, Emma J., Eves, Frank F., French, David P. and Hoppé, Roberta [62] | Cross-sectional study | N=200, Male=77.0%, Mean Age=20.1 (±3.72) years | United Kingdom | A series of hierarchical linear regression analyses were carried out with an intention to walk as the dependent variable. | Pedometer defined number of steps per day-week 2 | General Linear Models \| Hierarchical linear regression | Personal and psychological behaviour |
| Cook, I., Alberts, M. and Lambert, E. V. [9] | Longitudinal Study | N=151, 121 subjects (80. 1%), Mean Age=32.9 (± 10.97) BMI=26.0 (± 5.9) | South Africa | Investigated the association between adiposity and physical activity by taking adiposity measures as dependent variables. Explanatory variables forced into the model were mean steps day 1, age, motor vehicle access, completion of secondary school, comorbidities (self-reported diabetes and/or hypertension) and tobacco products usage. | Adiposity measures percentage body fat (PBF), body mass index (BMI) And waist circumference (WC) | General Linear Models \| Multiple linear regression | Health Related |
| Vander Ploeg, Kerry A., Maximova, Katerina, McGavock, Jonathan, Davis, Wendy and Veugelers, Paul [91] | Quasi-experimental design | Treatment Group(2009) =198 (Boys = 52.8%), Contolled Group (2009) =484 (Boys= 49.2%), Treatment Group (2011)= 196 (Boys=49.0%), Controlled Group = 309 (Boys=49.0%) | Canada | Assessed the relative inequity in physical activity and were compared between groups and over-time | step counts per day | General Linear Models \| Multilevel linear regression | Intervention |
| Craig, C. L., Cameron, C. and Tudor-Locke, C. [16] | Longitudinal Study | N=539 (out of 1,187 eligible children (Age range 5–19 years)), Boys=256 (Mean age=12.3 years) and Girls=283 (Mean age=11.4 years) | Canada | Associations between steps/day for parents and children (controlling for their age and sex) were estimated using general linear and logistic regression. | Steps/day-Son | Generalized Linear Models \| Logistic Regression | Family, Community and Sociodemographic |
| Inoue, Shigeru, Ohya, Yumiko, Odagiri, Yuko, Takamiya, Tomoko, Suijo, Kenichi, Kamada, Masamitsu, Okada, Shinpei, Tudor-Locke, Catrine and Shimomitsu, Teruichi [43] | Cross-sectional study | N= 790, Men=46.7%, Mean Age=48.3 (±13.7) years | Japan | Multiple logistic regression analyses were conducted to examine the relationships between sociodemographic variables and steps/ day | Relationship between steps per day and sociodemographic variables -[7500–9999 steps/day (vs <7499 steps/day)] for overall sample | Generalized Linear Models \| Logistic Regression | Family, Community and Sociodemographic |
| Kirunda, B. E., Wamani, H., Fadnes, L. T., Van den Broeck, J. and Tylleskar, T. [44] | Cross-sectional study | N=1208, women=50.1%, Aged ≤ 44 years=58.8%, Underweight = 7%, normal weight=67.8%, overweight= 17.8% obesity=7.4% | Peri-Urban and Rural Eastern Uganda | Identified factors associated with sedentary behavior and physical inactivity | Physical Activity -(Phyiscal inactivity (< 7500 steps/day averagely) And sedentary behavior’(< 5000 steps/day averagely)) | Generalized Linear Models \| Logistic Regression | Family, Community and Sociodemographic |
| McCormack, G., Giles-Corti, B. and Milligan, R. [47] | Cross-sectional study | n=603, 45% response rate, Men=205, Women=223 | Australia | Examined independent correlates of achieving ≥1O,000 steps/ day | continuous variable representing average daily pedometer step counts and a binary variable < 10,000 steps/day and ≥ 1 0,000 steps/day)-Men And Women | Generalized Linear Models \| Logistic Regression | Family, Community and Sociodemographic |
| Payn, T., Pfeiffer, K. A., Hutto, B., Vena, J. E., LaMonte, M. J., Blair, S. N. and Hooker, S. P. [15] | Cross-sectional study | N=85, Male=70.6%, Mean age = 57.9 years (±10.9) | Columbia | Compared the percentage of participants who met the PA recommendation across similar categories of mean daily steps (< 5,000, 5,001–9,999, >10,000) | meeting the average Steps/day recommendation | Generalized Linear Models \| Logistic Regression | Family, Community and Sociodemographic |
| Sigmundova, D., Sigmund, E., Badura, P., Vokacova, J., Trhlikova, L. and Bucksch, J. [48] | Cross-sectional study | N=278 parents, Mothers = 166, Mean Age=36.06 (± 4.28) ; N=194 (Their children) , daughters = 88, Mean Age=5.58 (± 0.84) | Czech Republic | Investigated whether parental achievement of the daily recommendation of step counts and non-excessive screen time on weekdays and weekend days (the two analyses separately) were associated with children’s achievement of daily step counts | Meeting the step count recommendation (11,500 steps/day) of the children | Generalized Linear Models \| Logistic Regression | Family, Community and Sociodemographic |
| Sisson, Susan B., Camhi, Sarah M., Tudor-Locke, Catrine, Johnson, William D. and Katzmarzyk, Peter T. [45] | Cross-sectional study | N = 3744, Men=46.8%, Mean Age=47.1 (±0.7) | United States of America | Determined predictors of sedentary classification (<5000steps/day) | Steps per day; body mass index(BMI); demographic, household and behavioral variables. | Generalized Linear Models \| Logistic Regression | Family, Community and Sociodemographic |
| Matthiessen, Jeppe, Andersen, Elisabeth Wreford, Raustorp, Anders, Knudsen, Vibeke Kildegaard and Sørensen, Mette Rosenlund [14] | Cross-sectional study | N=205, Mean Age=45.2 (±14.6) (2007–2008); N=1419 (2011–2012) Mean Age= 47.2 (±15.6) | Denmark | Continuous outcomes, i.e. steps/day, cadence and steps/day incl. cycling, were analysed using linear regression models; | pedometer measured PA (steps/day, cadence, step-defined activity levels, and steps/day incl. cycling) | Generalized Linear Models \| Logistic Regression | Family, Community and Sociodemographic |
| Villanueva, Karen, Giles-Corti, Billie and McCormack, Gavin [92] | Cross-sectional study | N=103, Female= 71 (69%), Private motor vehicle= 69 (67%) | Australia | Calculated odds ratios associated with achieving 10,000 steps/day by travel mode adjusting for gender, age, and LTPA. | achieving 10,000 steps/day by travel mode | Generalized Linear Models \| Multivariate Logistic Regression | Family, Community and Sociodemographic |
| Bailey, B. W., Borup, P., Tucker, L., LeCheminant, J., Allen, M. and Hebbert, W. [68] | Cross-sectional study | N=200 (young adult women), Age Range= 18–26 years. | United States of America | Assesses the odds of having excess body fat given the number of steps accumulated daily. | Relationship between steps per day and adiposity (body fat of greater than 32%) among college women | Generalized Linear Models \| Logistic Regression | Health Related |
| Newton, R. L., Jr., Han, H., Johnson, W. D., Hickson, D. A., Church, T. S., Taylor, H. A., Tudor-Locke, C. and Dubbert, P. M. [18] | Cross-sectional study | N= 379 African American adults, Mean age= 60.1 years, Female= 60% | United States of America | Assessed the relationship between categorical steps/day and the presence or absence of (1) MetS and (2) each of the five criteria components of MetS. | Presence of Metabolic syndrome criteria-MODEL 1 (adjusted for age and sex) And MODEL 2-(adjusted for age, sex, education, alcohol consumption, smoking and BMI) | Generalized Linear Models \| Logistic Regression | Health Related |
| Schofield, Grant, Schofield, Louise, Hinckson, Erica A. and Mummery, W. Kerry [37] | Cross-sectional study | N=415 (Australian adolescent girls), Mean age =16 (±0.8) year | Australia | Investigated relationships between physical activity (measured in steps per day), and CHD risk factors. | Physical activity of the participants depending on the CHD risk | Generalized Linear Models \| Logistic Regression | Health Related |
| Sisson, Susan B., Camhi, Sarah M., Church, Timothy S., Tudor-Locke, Catrine, Johnson, William D. and Katzmarzyk, Peter T. [38] | Cross-sectional study | N=1446, Men=48.2%, Mean age=47.5 years, Mean BMI=28.7 kg/m2 | United States of America | Determined if steps/day (categorical variable) was associated with odds of MetS and individual CVD risk factors. | Physical activty defined by Steps/day, Anthropometric and demographic measurements, fasting blood analyses and 24-hour dietary recall interviews | Generalized Linear Models \| Logistic Regression | Health Related |
| Park, H., Togo, F., Watanabe, E., Yasunaga, A., Park, S., Shephard, R. J. and Aoyagi, Y. [10] | Cross-sectional study | N=172, Female=96 (56%), Mean Age (Men)=73.0 (±4.4) years | Japan | Associations between bone health and the quantity and quality of habitual physical activity | risk of sustaining a fracture - Men | Generalized Linear Models \| Logistic regression | Health Related |
| Park, H., Park, S., Shephard, R. J. and Aoyagi, Y. [8] | Cross-sectional study | N=175 (Women=55%), Mean Age (years)=72.5 (±4.6), Mean Body mass index= (kg/m2)=23.6 (±3.0) | Japan | Assessed odds ratios and 95% confidence intervals adjusted for age, sex, smoking status (smoker/nonsmoker) and/or alcohol intake (drinker/nondrinker), and independent associations between habitual physical activity and the risk of sarcopenia. | Having appendicular sarcopenia | Generalized Linear Models \| Logistic regression | Health Related |
| Cook, I., Alberts, M. and Lambert, E. V. [9] | Longitudinal Study | N(Recruited)= 151, N (Completed)= 121 subjects (80. 1%), Mean Age=32.9 (±10.97) BMI=26.0 (±5.9) | South Africa | Investigated the risk of obesity in relation to pedometry assessed ambulation, odds ratios (ORs ±95% CI) were derived from logistic regression models for BMI-, WC- and PBF- defined obesity, adjusting for age, motor vehicle access, completion of secondary school, comorbidities and tobacco products usage. | risk of obesity-BMI-defined obesity (BMI>30kgm-2) | Generalized Linear Models \| Logistic regression | Health Related |
| Tudor-Locke, Catrine, Craig, Cora L., Cameron, Christine and Griffiths, Joseph M. [46] | Cross-sectional study | Boys= 5949 and Girls= 5709, Age range=5-19 years | Canada | Examined the associations between steps/day, parent-reported television watching time, and overweight and obesity status separately and combined (i.e., overweight/obese), controlling for child’s sex and age, and parent’s education level. The interaction between steps/day and television watching time was tested in each model. | Physical activity (steps/day) and parent's reported television watching time | Generalized Linear Models \| Logistic regression | Personal and psychological behaviour |
| Bennett, Gary G., Wolin, Kathleen Y., Viswanath, K., Askew, Sandy, Puleo, Elaine and Emmons, Karen M. [17] | Cross-sectional study | N=486, mean BMI= 29.8 (±7.2), Female=64% | United States of America | Association between reported hours of television viewing and pedometer-determined steps per day and whether television viewing was associated with the achievement of 10000 steps per day. | attainment of 10000 steps per day | Generalized Linear Models \| Logistic regression | Personal and psychological behaviour |
| McKercher, Charlotte M., Schmidt, Michael D., Sanderson, Kristy A., Patton, George C., Dwyer, Terenece and Venn, Alison J. [42] | Cross-sectional study | N=1995, Men=950, Mean age=31.6 (±2.6), Women=1045, Mean age=31.4 (±2.6) | Australia | Calculated prevalence ratios of depression and 95% CIs. | pedometer assessed steps/day and depression | Generalized Linear Models \| Log-binomial Regression | Health Related |
| Ablah, Elizabeth, Dong, Frank, Konda, Kurt, Konda, Kelly, Armbruster, Sonja and Tuttle, Becky [49] | Quasi-experimental design | Registrants= 2,515 , Participants= 1,292(51.4%), Female= 90% of registrants and 87% of participants. | United States of America | To predict the likelihood of success (achieving at least 70,000 steps) in each week following Week 1. | whether or not participants achieved 70,000 steps for that week. | Generalized Linear Models \| Repeated Measures Logistic Regression | Intervention |
| Moreira, C., Santos, R., de Farias Junior, J. C., Vale, S., Santos, P. C., Soares-Miranda, L., Marques, A. I. and Mota, J. [40] | Cross-sectional study | N= 417, Girls=243 and Boys= 174 boys, Mean age =16.5 (± 0.9) | Portugal | To verify the relationship between Metabolic Risk Factors (dependent variable in ordinal scale, and it was coded as 0 = no MRF; 1 = one MRF; and 2 = two or more MRF), with Physical Activity | Metabolic Risk Factors (dependent variable in ordinal scale, and it was coded as 0 = no MRF; 1 = one MRF; and 2 = two or more MRF) | Generalized Linear Models \| Ordinal logistic regression | Health Related |
| Park, H., Park, S., Shephard, R. J. and Aoyagi, Y. [8] | Cross-sectional study | N=175 (Women=55%), Mean Age (years)=72.5 (±4.6), Mean Body mass index= (kg/m2)=23.6 (±3.0) | Japan | Exponential regression analyses tested associations between habitual physical activity and muscle mass. | Degree of sarcopenia (age-related condition characterized by loss of muscle mass, with a concomitant decline of voluntary muscle strength and increase of fatigability ) | Generalized Linear Models \| Exponential Regression | Health Related |
| Al-Mohannadi, A. S., Farooq, A., Burnett, A., Van Der Walt, M. and Al-Kuwari, M. G. [55] | Longitudinal Study | N= 2088, Men=1390 and Women=698 | State of Qatar | Determined whether the demographic and climatic condition data were associated with daily step count. | step count/day | Multilevel Modelling \| Linear Mixed Models (LMM) | Weather and demographic |
| Chan, Catherine B., Ryan, Daniel A. J. and Tudor-Locke, Catrine [51] | Longitudinal Study | N=203; Men=25 , BMI = 28.7 ± 3.83 kg/m2, and Women=177 , BMI= 29.2 ± 5.92 kg/m2 | Canada | Examined the relationship between activity (log steps/day) and summarized daily weather-related variables | step count [Note:- The Type I p-value is associated with sequential tests of hypothesis and Type III p-value is associated with "last in" tests of hypothesis] | Multilevel Modelling \| Linear Mixed Models (LMM) | Weather and demographic |
| Smith-McLallen, Aaron, Heller, Debbie, Vernisi, Kristin, Gulick, Diana, Cruz, Samantha and Snyder, Richard L. [11] | Cluster-randomized trial | A total of 459 members enrolled in the program (234 in the enhanced condition and 225 in the standard condition). Participants ranged in age from 19 to 77 years, averaging 49.51 years (SD, 11.12) at baseline, and 56% were female. | United States | Examined the effect of condition on daily step counts while accounting for the clustering of participants within intervention group | Daily pedometer step counts (steps/day) | Multilevel Modelling \| Mixed model | Intervention |
| Reznik, Marina, Wylie-Rosett, Judith, Kim, Mimi and Ozuah, Philip O. [52] | Randomized Controlled Trial | N=500 (control), Mean age=5.1 ; N=488 (intervention), Mean age=6.9 . | United States of America | Hierarchical linear models were fit to evaluate differences in mean number of steps between the two groups. | Pedometer defined number of steps per school day | Multilevel Modelling \| Generalized Linear Mixed Model | Intervention |
| Chapman, Gretchen B., Colby, Helen, Convery, Kimberly and Coups, Elliot J. [72] | Randomized Controlled Trial | Study1- N= 148, Mean Age= 48 y, Women= 125, Non-Hispanic white=60% African American= 16% , Asian14% , Hispanic= 6% , with the remainder other races or multiple races; Study2- N= 64, Women= 60; Mean age= 45 y [Age range, 23–69 y], non-Hispanic white= 72%, Asian= 11% African American= 9% and Hispanic= 8% | United States of America | Mixed models that include experimental condition, day of the intervention and baseline average | steps walked per day as recorded in the pedometer memory. | Multilevel Modelling \| Mixed Model | Intervention |
| Arigo, Danielle, Butryn, Meghan L., Raggio, Greer A., Stice, Eric and Lowe, Michael R. [53] | Longitudinal Study | N=294 , Mean Age=18(±0.44), Mean BMI =23.65 kg/m2 (±2.88). | United States of America | Relations between body image/perceptions of eating behavior and pedometer-assessed PA over the first 2 years | Physical activity measure by the steps/day | Multilevel Modelling | Health Related |
| Bennett, Gary G., Wolin, Kathleen Y., Viswanath, K., Askew, Sandy, Puleo, Elaine and Emmons, Karen M. [17] | Cross-sectional study | N=486, Mean BMI= 29.8 (±7.2), Female=64% | United States of America | Association between reported hours of television viewing and pedometer-determined steps per day and whether television viewing was associated with the achievement of 10000 steps per day. | Effect of Reported hours of television viewing on Steps per day | Multilevel Modelling \| Mixed Model | Personal and psychological behaviour |
| Bennett, G. G., McNeill, L. H., Wolin, K. Y., Duncan, D. T., Puleo, E. and Emmons, K. M. [56] | Randomized cluster design | N=1735, Mean Age= 49, Mean BMI=30.0, Female= 1,270 (73.2%) | United States of America | Regression equations predicting steps/day where the independent variable of interest is perceived neighborhood safety | Steps per day | Multilevel Modelling \| Random effect Regression | Family, Community and Sociodemographic |
| Sniehotta, Falko F., Presseau, Justin, Hobbs, Nicola and Araújo-Soares, Vera [54] | Randomized Controlled Trial | N= 10 (normal and overweight adults) , Femal=6, Males=4, Mean BMI=25.4 (±1.99), Mean Age=36.9 years (±17.46) | Scotland | The main effects of each condition ( step-count goal prompt vs. alternative goal prompt And self-monitoring: open vs. blinded pedometer) between and within participants were tested | Daily pedometer step counts over 60 days | Multilevel Modelling \| Random Intercept multilevel model | Intervention |
| Ling, Jiying, Robbins, Lorraine B., McCarthy, Valerie Lander and Speck, Barbara J. [61] | Longitudinal Study | N= 133, Mean Age= 9.26 (±0.93), Mean BMI= 20.74 (±5.12), Sex (female)= 59 (44.4%) | United States of America | Estimated the direct and indirect effects of self-efficacy, enjoyment, parental influence, and environment on self-reported PA and pedometer steps | pedometer assessed steps/day | Structural Equation Modelling \| Path Analysis | Personal and psychological behaviour |
| Seghers, J., Vissers, N., Rutten, C., Decroos, S. and Boen, F. [20] | Cross-sectional study | N= 1272, Boys= 547 and Girls= 725, Mean age= 11.42 years (±0.47). | Belgium | Structural equation modelling was performed to test the hypothesized motivation sequence and to examine the mediation effect of autonomous and controlled motivation towards leisure-timesport and PA on the relation between goal content and steps per day. | Motivational and Behavioural outcomes with Physical activity (objectively meausred step count) | Structural Equation Modelling | Personal and psychological behaviour |
| Ramirez, E., Kulinna, P. H. and Cothran, D. [64] | Cross-sectional study | N= 479, Mean age= 9.8 yrs, Male= 50.1% , Mean= 19.37 (±4.18) | United States of America | Determined associations between self-efficacy and physical activity participation. | Physical activity participation measured by steps per day | Structural Equation Modelling | Personal and psychological behaviour |
| Standage, Martyn, Gillison, Fiona B., Ntoumanis, Nikos and Treasure, Darren C. [60] | Longitudinal Study | N= 494 (secondary school students), Mean age = 12.58 years (±0.74), Boys= 201, Girls= 291, Gender not specified= 2 | England | Examined the adequacy of the proposed model of autonomous motivation toward exercise will positively predict health-related quality of life (HRQoL), physical self-concept (PSC) and the number of steps taken over a 4-day period (H4). | Health-related quality of life (HRQoL), Physical self-concept (PSC), and 4 days of objectively assessed estimates of activity. | Structural Equation Modelling | Personal and psychological behaviour |
| Castillo, Jacqueline C., Clark, B. Ruth, Butler, Carling E. and Racette, Susan B. [63] | Cross-sectional study | N=212; Mean age= 9.9 years, 81.7% black, In Grades 2–5 attending urban public schools with high eligibility for the National School Lunch Program. | United States of America | Generalized estimating equations with an exchangeable correlation matrix were used to compare total in-school steps and MVPA steps between PE and non-PE days and to explore sex differences | In school total physical activity measured by pedometers and Cardiorespiratory fitness measured using 20-meter Progressive Aerobic Cardiovascular Endurance Run | Generalized Estimating Equations | Intervention |
| Smith-McLallen, Aaron, Heller, Debbie, Vernisi, Kristin, Gulick, Diana, Cruz, Samantha and Snyder, Richard L. [11] | Cluster-randomized trial | N= 459, (N=234 in the enhanced condition and N= 225 in the standard condition). Age Range = 19 - 77 years, Mean Age= 49.51 years (±11.12) at baseline, and Female=56% | United States of America | Generalized estimating equations were used to model the average number of steps logged at each study period. | Participation- (Percent of Days Logged for Those Who Logged Any Days) | Generalized Estimating Equations | Intervention |
